# Impact of commercial over-reimbursement on hospitals: the curious case of central Indiana

**DOI:** 10.1007/s10754-018-9249-9

**Published:** 2018-09-06

**Authors:** Michael F. Seibold

**Affiliations:** Seibold and Associates, Tucson, AZ USA

**Keywords:** Hospital reimbursement, Hospital costs and efficiency, Indiana, I110, G22

## Abstract

An employer coalition in Indiana sponsored a study by the Rand Corporation examining commercial insurer payments as a percent of Medicare. The employers sought to understand why their health care costs were high and increasing. The study showed that, on average, their insurer was paying three times what Medicare pays for the same services. In this, a follow-up study, we demonstrate that these high payments resulted in very high profit margins for central Indiana’s major health systems, along with elevated costs and poor performance on key efficiency measures. We also see indications that hospitals appear to be using aggressive revenue cycle management techniques. The paper concludes with a discussion of policy issues.

## Background

A client asked our firm to examine the impact of information contained in a Rand Corporation report sponsored by an Indiana employer coalition. The report examined what employers in central Indiana were paying hospitals relative to Medicare payments for the same services.^1^

Over the past decade, my firm has evaluated the performance of contracts between more than a dozen health plans and hospitals. The goal of these engagements is to negotiate contracts that are “fair” to both parties, i.e., ones that will generate enough money to sustain the hospitals over time, promote both quality and efficiency, and serve as the best solution to each community’s health care cost problems.

Generally, these health plan and health system negotiations result in an agreement acceptable to both sides. Occasionally, however, negotiations result in bruising public fights between hospitals and health plans. Sometimes the data we develop show that a hospital or health system is being underpaid; in such cases, if the data show that the hospitals are efficiently run, margins are unsustainable, and/or the health plan has gone too far in pushing down prices, we advocate for increased payments for those health systems.

In the Rand study, we found the pricing numbers very high. The clients we have worked with around the US, typically the largest health plans in each market, pay much lower rates as a percent of Medicare, usually in the range of 145–180%—roughly two-thirds of what the insurer in the Rand study seems to be paying. At these lower rates, hospitals are quite capable of generating sufficient margins; non-profit hospitals have historically averaged about 2–3% total margins.^2^ These margins are more than adequate to fund ongoing capital requirements, equipment purchases, and investments in technology and other areas of the business.

After the 2008 recession, health care costs and prices moderated; now, a decade later, they are much higher and inflating rapidly. Recent studies^3^ identify price increases, not utilization or demographics, as the driver. Understanding pricing has become an important policy issue nationwide.

Our client, concerned about the implications of the Rand study, engaged us to explore the impact of commercial payment levels on Indiana hospitals. However, we also needed to think about what could generate these results. Here are some potential causes in no particular order:

1. *Market concentration* Distribution of power among market participants may be a factor. Typically, a market participant with monopoly power can demand higher payments. For example, a dominant academic medical center with a monopoly on highly advanced treatments may use its power to increase reimbursement even when there are many other hospitals in the market. Because the health plan must include that provider in their network, they are forced to meet the health system’s price demands. These “monopoly rents” may drive up costs significantly.
2. *Management competence* We expect managers to be rational. However, managers may ignore market signals and relevant data when evaluating contract performance. In our work over the past decade, we have been surprised at how managers in health plans have simply not looked at critical data to hold health systems accountable for their prices. This includes calculating actual rates of increase in unit prices compared to what was negotiated in the contract; hospital profits and contribution margins (what insurers pay over and above the health system’s costs as shown on their cost reports); and costs and efficiencies compared within and across markets. Despite an abundance of publicly available data, such analyses are not routine in our experience.
3. *Distributor (broker and benefits consultants) behavior* If one examines requests for proposals from major benefit consulting firms, one rarely finds affordability as a key driver of decision-making. Rather, the primary focus of the competitive financial analysis is differences among carriers in terms of discounts and administrative fees, whether or not their payment levels make sense. How do payments compare to Medicare? Do these rates result in either outsized hospital profits or serious losses?
4. *Focus of negotiations* Following the distributors’ lead, health plans tend to focus on discount and fee levels rather than affordability. In fact, it is hard to find evidence of a focus on affordability in health plan or health system mission or vision statements. One would imagine that affordability should be a primary goal.
5. *Financing* While the preponderance of firms are small groups with fewer than 500 employees, large groups, with most of the employed population, wield the most market influence. Most firms above 100 employees have an “administrative services only” contract with the health plan, perhaps with reinsurance, taking most or all of the risk from the insurer. The insurer, therefore, has less incentive to control claims costs via tougher provider contracts since this get passed directly to the employer and member.
6. *Employer behavior* Employers, particularly large ones that can impact a market, may be part of the problem. They seem to want to lower their cost of care but are reluctant to take on bolder initiatives such as:
  - supporting health plans who want lower prices by promising more volume to a health system in return for lower prices, because employers do not want to make employees change providers.;
  - engaging their insurers, providers and regulators to find solutions to high costs from a community-wide perspective—including public support for health plans if providers cancel contracts over unreasonable price demands;
  - chasing “the next big thing” touted by benefits consultants that really do not address the core issue of pricing: point of service plans; HMOs; case and disease management programs; “consumer directed” high deductible health plans; and wellness programs. While some of these programs (e.g. wellness) are clearly the right thing to do, and may provide tactical benefits for insurers (e.g. using higher cost sharing levels to reduce an employer’s premiums without really affecting underlying costs), collectively they have not substantially reduced growth in health care expenditures.
7. *Regulation* In reaction to the perceived abuses of the health insurance industry by health plans in the 1990s and 2000s, states have instituted a number of laws and regulations. Among the many restrictions are “any willing provider” laws, which require the insurer to contract with any provider willing to agree to the price and other contract requirements. Other regulations include mandates for paying out-of-network claims, which may result in out-of-network providers being paid at levels higher than network providers, thereby reducing the impact of small network products; and limits on utilization management programs (e.g., 1-day lengths of stay for normal deliveries, limitations on denials, etc.).

We will not be exploring all of these potential contributors to high prices. However, we believe these data do provide support that validates some of these hypothetical contributors.

## The data

We used Medicare cost report data^4^ (2012 and 2016) for 31 non-profit hospitals in central Indiana, including greater Indianapolis, Lafayette and Muncie. Given that the Indianapolis market has consolidated both horizontally and vertically over the past decade, where necessary we combined the data of hospitals that operate within systems in order to more accurately assess overall health system performance. In one case, when we had questions about a system’s cost reports, we reviewed their published financial statements.

For comparison purposes, we drew on two projects for which we had comparison cost report data for the same time periods so that we could examine both a single point in time and changes over that period. This is not a statistical study, so we do not posit this data as statistically representative. We selected Chicago and a market in New York State; because both operate in presumably higher cost environments, they make good comparison markets for this study.^5^

We examined several variables from the Medicare reports:

- Net patient service revenue
- Total margin
- Medicare margin (both before and after Disproportionate Share and Graduate Medical Education payments)
- Charity and bad debt expense as percent of revenue
- Case mix index
- Utilization
- Occupancy
- Inpatient cost per Medicare discharge
- Supply cost per discharge
- Salaries per discharge
- Full time equivalent staff per occupied bed (both direct and overhead)

Not all these variables are reported here.

Commercial insurance payments as a percent of Medicare are a function of both the commercial payment numerator and the Medicare payment denominator. Medicare reimbursement rates vary by state and city. If Medicare payments in central Indiana are lower than other locations, then commercial rates can be expected to be higher as a percent of Medicare.

**Fig. 1:**
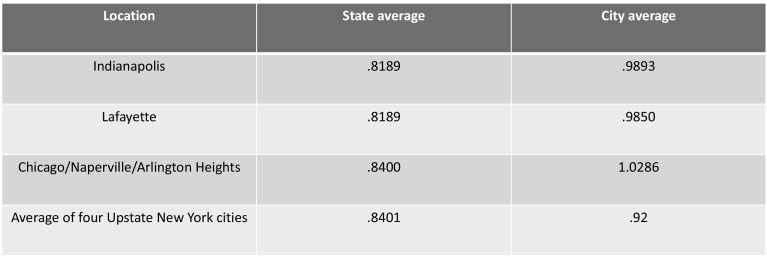
2016 Wage Indexes for selected markets. *Source*: CMS. Downloaded table: CMS-1632-Final-Correction Notice Table 3. Wage Index file

Medicare payment rates are adjusted geographically for wage differences. Wages are the single largest expenditure in hospitals so it makes sense to adjust rates for wages. We compared both the state level and city level wage indexes (Fig. 1) to determine if variation in wage indexes could explain the high commercial payment rates relative to Medicare. The US average is 1 and each location index is expressed relative to that number. A low wage state might have an index of .7 while a high wage state like California has an index of 1.3. Some city indexes such as San Francisco exceed 1.7 meaning their wage levels are 70% higher than the US average.

The wage index data indicates that Medicare payment rates do not explain the high commercial payment levels relative to Medicare due to the modest differences among locations.

**Fig. 2:**
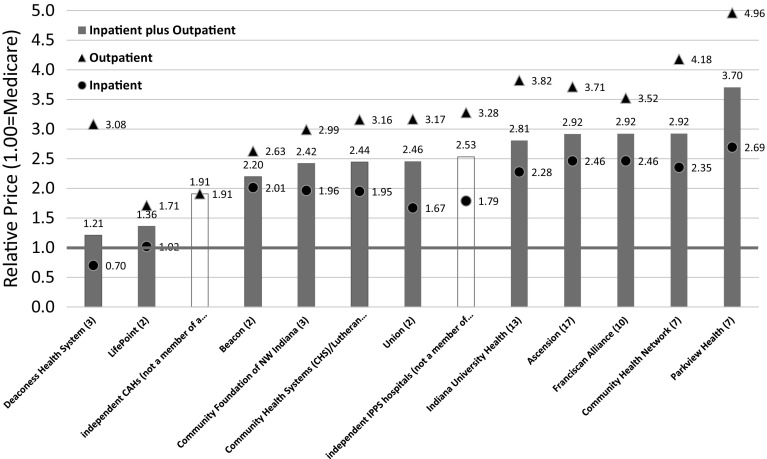
Rand analysis–relative prices of Groups of Hospitals. White Chapin. Hospitals pricees in Indiana: Findings from an Employer-Led Transparency Initiative. Santa Monica, CA: RAND Corporation 2017. https://www.rand.org/pubs/research_reports/RR2106.html. Hospital systems are shown in solid bars, independance are clear. This analysis is based on claims from self-funded employees for services rendered at community hospitals for the period 7/2013 through 6/16

Figure 2 presents the Rand data on Indiana payment rates as a percent of Medicare—about three times what Medicare pays. Furthermore, for outpatient services, the top four systems average 3.8 times Medicare.

## Are these payment levels high?

Because contracts between health plans and hospitals include confidentiality clauses, it can be difficult to acquire useful data on commercial payments compared to Medicare. Although health plans often derive such figures from their own and Medicare data, they are unable to compare rates to those of other health plans. Furthermore, despite that fact that self-funded employers have access to their own claims data, it appears that neither they nor their benefits consultants examine payment as a percent of Medicare when evaluating health plan contracting effectiveness. As noted previously, they focus on favorable payment rates among commercial payers in evaluating total cost of care. This may explain why the Indiana employer group resorted to a national research firm to collect and evaluate the data.

Distributors do not ask for this data either, in our experience. Again, the focus appears to be on discount and fee differences and not on the reasonableness of rates. Since RFPs for larger employers cover multiple states, one would think that consultants and brokers would focus on differences in comparative payment rates among locations. This does not appear to be the case.

In our consulting projects, we typically see average commercial payment levels of between 140 and 200% of Medicare. In projects where we have acquired data for all payers, we find that the largest insurer typically enjoys a payment advantage of 10–20% on hospital services.

**Fig. 3:**
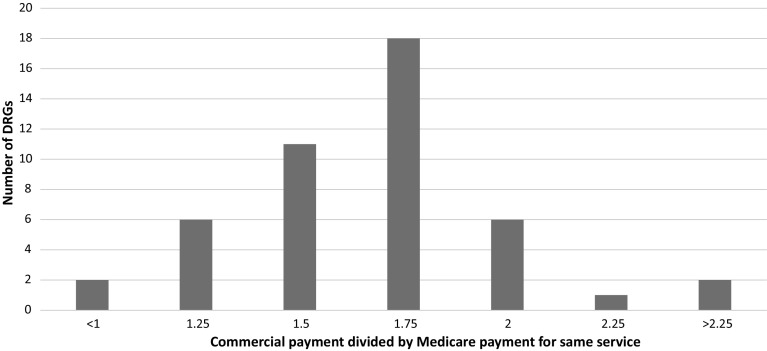
2012 Commercial-to-Medicare FFS payment ratios for 50 DRGs. *Source*: America's Health Insurance Plans. National Comparisons of Commercial and Medicare Fee-For-service Payments to Hospital, Data Brief, 2016

America’s Health Insurance Plans (AHIP) completed a study comparing commercial payment rates for 50 high-volume DRGs.^6^,^7^ Of these, 78% were at or below 175% of Medicare. However, these ratios do not include patient liability data. Factoring in patient liability (roughly 5–10% for traditional Medicare), the ratio increases to 175–185% of Medicare—a figure consistent with data from specific markets we have studied (Fig. 3).

## Impact of high payment rates on hospitals

We wanted to examine the impact of these apparently high prices on central Indiana hospitals. Theoretically, if an organization is being overpaid, several outcomes are possible, none of which are mutually exclusive. Such an organization is likely to:

1. Sustain higher profits, higher retained earnings, and capital
2. Pay staff and suppliers more than do lower-margin health systems
3. Be less efficient operationally in terms of managing supplies and other costs
4. Add unnecessary capacity, specialty services or technology
5. Invest in quality and population health improvement activities
6. Use excess revenue to expand either vertically (e.g., owning physicians, starting their own health plans) or horizontally (mergers)
7. Greater provision of community benefits such as charity care

We have not explored any of the data on quality or community benefit activities of central Indiana health systems.

## Payment rates and profitability

**Fig. 4:**
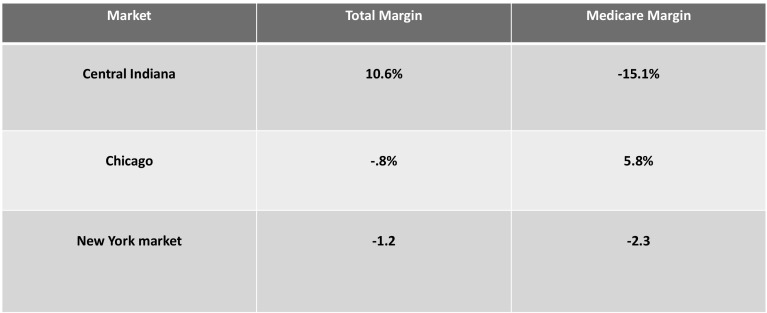
Median hospital margins: overall and Medicare. *Source*: CMS cost Reports

One would expect that high commercial payment rates would result in higher-than-average profitability. This is exactly the case in Indiana (Fig. 4).

Compared to the US average for non-profit hospitals, all four of the major Indiana systems had high margins. Each of the four systems shows margins in excess of 10%, with the Franciscan Alliance system at 20%. While IU Health shows an overall loss on their cost reports, this loss is not consistent with their audited financial statements, which indicate a much higher margin.^8^ If we use IU Health’s audited financial statements less a charge to its income statement from a divestiture, the market median would exceed 12%.

Not surprisingly, non-system hospitals averaged a loss of 15% overall (9.7% on Medicare), likely reflecting their relative lack of bargaining power and resulting lower payment rates. Non-system hospitals have lower occupancy rates. Since hospitals have high fixed costs, low occupancy means there are fewer patients over which to spread costs. In addition, lower occupancy in non-system hospitals could be due to system hospitals employing most of the physicians in the market, reducing referral opportunities for non-system facilities. According to the Rand study, these hospitals received lower commercial payments.^9^ The data would indicate that the larger health systems have stronger bargaining positions with this insurer.

While Indiana hospitals are very profitable overall, many lose a significant amount on Medicare. Research indicates that high payment rates by commercial insurers lessen the pressure for health systems to manage government program costs.^10^

**Fig. 5:**
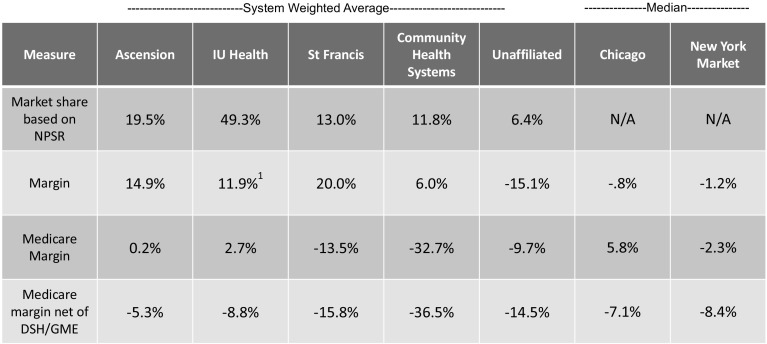
Selected financial measures by system. *Source*: CMS Medicare cost reports; IU Health Audited Financial Statements, 2016.^1^From audited financial statement. See footnote 8, page 7

Weighted averages for each system and unaffiliated hospital appear in Fig. 5. These were weighted by net patient service revenue.

The total margin includes Medicare, Medicaid, commercial health plan payments, and private-pay patients. The Medicare margin applies only to traditional Medicare, not Medicare Advantage or Medicaid. Hospitals tend to lose a lot on Medicaid because reimbursement is typically much lower than Medicare. Thus, the commercial contribution to profit margin (the amount paid by the insurer to the hospital, divided by allocated hospital costs) is likely to be quite high. We suspect that commercial payment levels to central Indiana hospitals could be decreased by at least one-third while still leaving hospitals with margins adequate to sustain these health systems. Such a decrease would also encourage efficiency improvements that help reduce long-term demands for higher reimbursement.

Compared to other markets and US averages, central Indiana’s major non-profit health systems are exceptionally profitable. However, unaffiliated hospitals are losing significant amounts of money, both overall and on Medicare.

What about costs? Has the higher reimbursement to Indiana hospitals allowed them to be less than aggressive with managing costs?

An “apples to apples” comparison we use in the cost report data is the “inpatient Medicare cost per discharge,” which applies only to the allocated costs of inpatient confinements for Medicare patients. In our research we typically use the hospital’s case mix index—a measure of patient acuity that is generated from hospital claim coding—to control for patient acuity mix to make hospital to hospital comparisons. Hospitals with sicker patients should have higher costs.

**Fig. 6:**
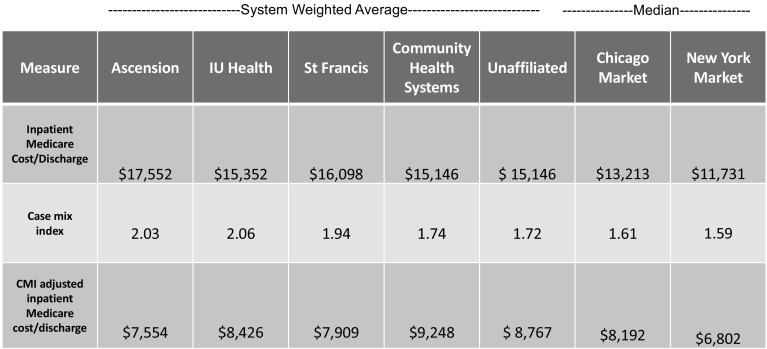
Cost per inpatient Medicare discharge and case mix indexes. *Source*: CMS Medicare cost reports (2016)

Figure 6 shows the raw costs, the average case mix index for each system, and comparisons to the Chicago and New York markets.

The data show that for unadjusted inpatient cost per discharge, central Indiana hospitals have considerably higher costs per discharge. When adjusted for CMI, they are closer to Chicago’s figures, but are still higher than the market in New York. However, Indiana case mix indices appear very high. We wanted to know why.

## Case mix index and utilization: central Indiana coding practices

**Fig. 7:**
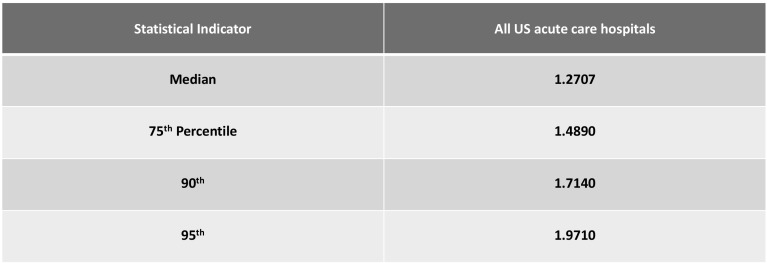
Case mix index statistics for all US acute care facilities, 2015. *Source*: CMS. 2015 Case Mix Indexes for all US acute care hospitals

Figure 7 offers information on case mix indices for central Indiana, the US, and our two comparison markets.

Case mix indices are a function of Medicare Severity-Diagnosis Related Groups (MS-DRG) coding by each hospital. Hospitals devote a great deal of effort and expense to “revenue cycle management,” processes that maximize reimbursement for any given patient, presumably within CMS coding guidelines. Indiana hospital claim coding generates very high case mix indices. We believe most insurers in Indiana are using the DRG system for inpatient cases although some may still be using discount off charges for outpatient services.

A high case mix for a given hospital may be a function of a number of factors:

1. Since cost report CMIs apply only to traditional Medicare patients, a hospital could experience high CMIs if a significant cohort of healthier patients disproportionately enrolled in Medicare Advantage plans.
2. Patients may be sicker overall than in comparison markets.
3. Hospitals may vary in how aggressively they have implemented “revenue cycle management” programs to maximize reimbursement for any given patient.

We wanted to get a sense of these hospitals’ Medicare case mix indices as compared to national data.

Indiana case mix indices are higher than the US average (Fig. 7). Every hospital in the sample exceeded the 90th percentile of all US acute-care hospitals on average, with IU Health and Ascension above the 95th percentile. These case mix indices would be reasonable in an academic medical center that treats very complex cases. IU Health has both a children’s and an academic medical center, and we would expect high case mix indices in those two. But IU Health also has a number of community hospitals in its system. But because IU Health is made up of both academic and community hospitals this CMI appears high. The other IU Health hospital CMIs are above the 75th percentile of all US hospitals.

Could Indiana patients just be sicker than these other markets? The answer is, generally yes.

**Fig. 8:**
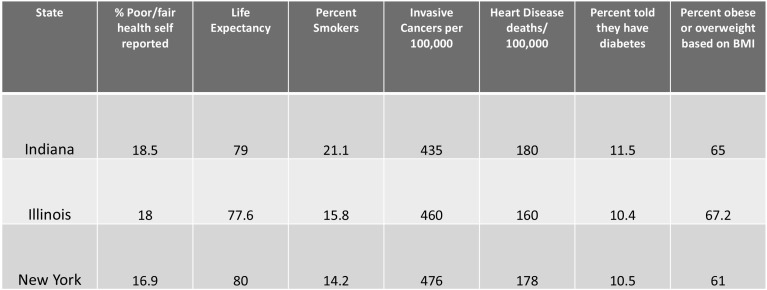
Selected population health statistics. The Kaiser Family Foundation State Health Facts. *Data Source*: The Centers for Disease Control and Prevention (CDC), National Vital Statistics Reports (NVSR), Vol. 66, No. 1: Births: Final Data for 2015, January 5, 2017

Figure 8 presents selected population health statistics. Indiana residents have a lower life expectancy and higher rates of obesity, heart disease and diabetes than the comparison markets. Again, as in most of the US, Indiana’s significant health care spending does not necessarily result in a healthier population (Kaiser Family Foundation 2017).

Clearly, Indiana residents are in poorer health. It is difficult to estimate the impact of this on CMIs; however, if CMIs were increasing over time at a high rate, then we would get a sense of how much is due to poor health versus coding practices.

In 2012 and 2013, Centers for Medicare and Medicaid Services (CMS) implemented an annual reimbursement adjustment of − .2 per year. CMS was (correctly) anticipating increased coding intensity due to the implementation of the 2008 Medicare Severity-Diagnosis Related Group MS-DRG) system. CMS went on to further reduce reimbursement by .8 per year from 2014 through 2016.^11^

**Fig. 9:**
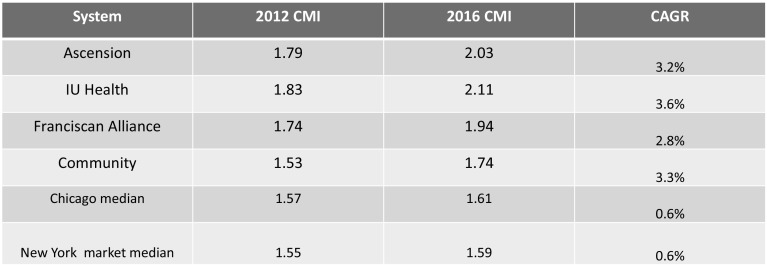
Median case mix indices 2012–2016. *Source*: CMS Cost Reports

Figure 9 shows that central Indiana hospitals increased their already-high CMI coding levels dramatically from 2012 to 2016, well above what CMS has engineered into the prospective payment system to account for anticipated increases in coding intensity.

As we mentioned previously, in recent years hospitals have invested heavily in “revenue cycle management” programs, which are focused on maximizing reimbursement from MS-DRG reimbursement system, presumably within guidelines to prevent fraud. Claims are periodically audited by Medicare to ensure CMIs are supported by the medical record.

The large rate of increase in these hospital’s CMIs is troublesome. It is not explained by the relatively poor health status of state residents, as these patient statistics are fairly consistent over time.

Another possible explanation is potentially adverse selection by Medicare Advantage Plans. Because these are considered commercial insurance plans, they are reported with commercial plans in cost reports. Thus, if the Medicare Advantage population is much healthier, it would leave the sicker patients in traditional Medicare, potentially explaining the higher Medicare CMIs. For example, a healthy senior, anticipating little need for care and unconcerned with who is in their network, is likely to select a lower-premium, narrower-network Medicare Advantage plan. Alternatively, if they are seasonal residents, they may find traditional Medicare a better fit for their lifestyle, as it covers services anywhere within the US.

Marion County’s (Indianapolis) senior population has been moving toward Medicare Advantage enrollment, which has doubled between 2012 and 2017.^12^ Medicare Advantage members represent 39% of Marion County’s over-65 population, while only 30% of Cook County’s seniors are enrolled.^13^ Can this variance explain the sizable difference in case mix indices?

**Fig. 10:**
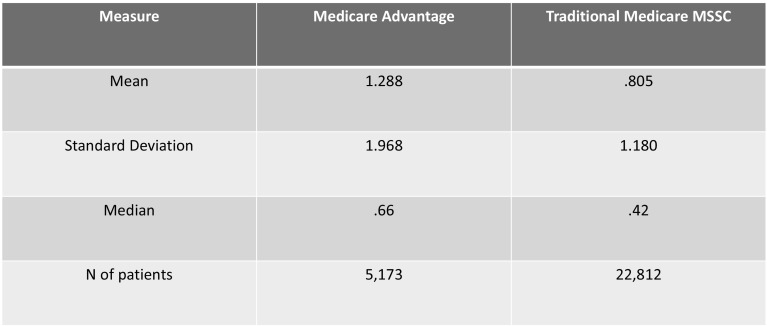
Risk adjustment scores for traditional Medicare and Medicare Advantage patients. *Source*: American Health Network data; Data based on year ending 12/31/2017

We examined our client’s Risk Adjustment Factors^14^ for Medicare Advantage patients compared to Medicare patients enrolled in a traditional Medicare to see if Medicare Advantage patients were healthier than traditional Medicare in an Accountable Care Organization.^15^ Figure 10 includes these data. As the chart demonstrates, Medicare Advantage patients in this cohort are in fact sicker than those in traditional Medicare. Thus, the cause of the high case mix indexes is not adverse selection.

Based on this analysis, we think that case mix adjusted data should not be used in the cost comparisons, and therefore conclude that high payment rates by commercial insurers are contributing to high costs.

To recap:

1. Indiana health systems are paid at much higher rates compared to the other markets and AHIP data.
2. Those payment levels result in outsized profits.
3. Indiana providers have high costs.
4. Indiana health systems have high case mix indices, which appear unrelated to the actual health status of Indiana residents.

## Efficiency

**Fig. 11:**
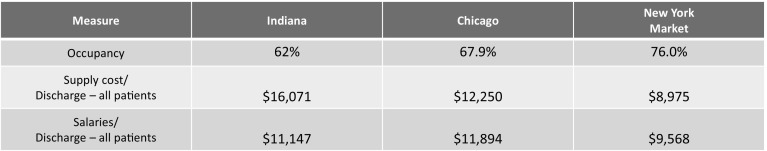
Weighted (by NPSR) efficiency measures. *Source*: CMS cost reports (2016)

We examined a number of efficiency measures in the cost report data (Fig. 11).

Ideally, hospital occupancy levels should meet or exceed 80% for optimal efficiency while meeting the seasonality demands.^16^ Nationally, some markets and major academic medical centers routinely achieve these levels or even higher, but few community hospitals do. As seen in Fig. 11, Indiana has excess capacity. Some Indiana facilities have occupancy rates below 50%.

In addition, Indiana hospitals have high supply costs per discharge. High supply costs can be a function of various possible issues: poor inventory management; the effectiveness of group purchasing activities; and/or allowing physicians unrestricted choices regarding implants and other devices (rather than limiting choices to a few versions to control costs).^17^

Finally, salaries in Indiana appear high relative to comparison markets. One would not expect the cost of living to be as high in Indiana as it is in Chicago and New York.^18^

The poor efficiency levels in these hospitals may be further indicators that high commercial reimbursement levels significantly reduce the incentives for health systems to contain their costs across multiple areas of their organizations.

## Discussion

The State of Indiana prides itself on offering a business-friendly, low-regulation environment. Decades ago, the state abandoned its “certificate of need” process which some states have used successfully as a capacity control mechanism on health providers. Since that time, the state has allowed essentially unfettered development of the health care market. A byproduct has been excess capacity, with no apparent let-up in development: at the time of this writing, Indiana had 16 new health facility projects in development, each with costs in excess of $32 million.^19^ A billion-dollar development project for a new facility on Indianapolis’ north side was recently announced, in a market where occupancy rates are already low.^20^

Insurers estimate that today, over 70% of Indiana physicians are employed by hospitals; the national average is about 50%.^21^ Per-physician acquisition costs can vary from $75,000 to over a million dollars, depending on specialty, patient panel size, and expected referral volume. The Medical Group Management Association estimates that nationally, hospitals lose between $75,000 and $200,000 per employed physician per year.^22^

However, it is the excess payments from health plans that have enabled this situation to develop. Without excess reimbursement by commercial health plans, this could not have happened absent another source of income.

It is difficult to understand why this insurer allowed prices to rise to this level relative to Medicare. There may be a number of reasons: unwillingness to use the kind of data we have discussed here for negotiations; the risk of public battles during hospital contract negotiations impacting the insurer’s brand; lack of support from employers in public battles or for smaller network products, which would provide opportunities to negotiate lower payment rates for anticipated or guaranteed shifts in volume; or not defining the problem as an affordability issue for the community. Regardless of the reasons, these employers should be asking questions of this insurer.

As health systems continue to consolidate and employ more and more physicians, negotiating power has shifted strongly in favor of providers. Further, insurers experience ongoing issues negotiating rates in non-competitive markets with “sole source” providers such as academic medical centers and children’s hospitals (naturally, most patients are reluctant to be referred to facilities outside their community).

What to do?

## Local to Indiana

The Indiana employer coalition appears to be motivated to engage health plans, providers and state government to address high benefit costs, and have engaged a prestigious consultant to help them. It may be in the employer group’s best interest to use the data we have presented here to challenge hospitals and the health plans about their behavior, setting targets for reimbursement and cost reductions and holding the health systems accountable for results. They might wish to consider leading a community-wide dialogue to encourage health systems to add affordability, accountability, transparency, and efficiency to their organizational goals. To gain the attention of the health systems and ensure success, the coalition would need to demonstrate that they are willing to make changes such as:

1. Supporting their health plans in eliminating over-reimbursed high cost health systems who will not commit to change,
2. Requiring the insurer to set up a bidding process in which the four systems submit bids based on total cost of care. This creates the opportunity for real competition among the systems. Be firm with the systems that if only one system responds, then they will get all of their business.
3. Requiring a 15% initial reduction in the major insurer’s rates and a 3% decrease per year for 5 years as “table stakes”, to allow the systems time to reduce their operating costs as pricing is reduced. If employers want to offer all the systems, then premiums must be tied to each systems’ bid price. Alternatively, an employer could offer a plan involving only the lowest-cost system.
4. Changing benefit plans to support movement of patient volume to lower cost systems,
5. Insisting their insurers not use antiquated reimbursement methods such as discount off charges, and that incentive programs are externally audited to ensure they are generating value, not just additional income for providers,
6. If health plans are not responsive, moving to a third-party claims administrator and collectively building their own custom network. This would put tremendous pressure on the health plans by using the competitive market the way it should be used: to create competition in a highly consolidated insurer market,
7. Engaging the state on regulatory actions to reduce capacity and costs.

## National policy implications

Indiana is not alone. The history of US health care over the past 50 years is one of missed opportunities and rising costs, which are now approaching 20% of GDP. Compared to OECD countries, we pay significantly more on a unit cost basis for health care, and have poorer outcomes.

Some have called for “Medicare for All” or at least “Medicare Advantage for All,” which would marry a government program (Medicare) to private insurer administration (Medicare Advantage) and make government-sponsored health plans available to a larger population. However, the data suggest the challenges that Medicare Advantage plans would face if it were opened up to a larger population.

Medicare Advantage plans typically have health system and physician payment rates close to those of Medicare. Medicare is a monopsony in terms of its ability to impose its prices on the market, and health plans were able to tie into that pricing power by arguing that hospitals would essentially get the same payment from traditional Medicare. The health plans could promote their incentive programs for cost and quality measures and aggressively code RAF scores to increase reimbursement, making the program even more attractive to health systems.

However, expanding Medicare Advantage to a larger population points out the disconnect between government and commercial pricing. What would health systems do if they no longer received these excess payments to cover their high costs? This is the challenge for any potential universal health insurance program in the US.

The larger question is, can markets solve this problem, or are more aggressive actions required on the part of government? By default, health plans are meant to control commercial reimbursement increases, but absent the regulatory power of the government to impose pricing, the current outcome should be no surprise.

Employers have shifted risk to their employees over the past decade through higher deductibles, copayments, and out-of-pocket limits and premiums. These actions have not stopped the trend toward higher premiums, other than the one-time impact of the benefit reduction to the premium.

Health care costs already consume a significant portion of the median household’s after-tax earnings. This would indicate that consumer-driven price elasticity strategies are a fool’s errand, and one with potential iatrogenic effects. If an insurer with 70% market share has not been able to address the cost problem, how can an individual consumer? The current system is too opaque, and fraught with competing interests and goals, none appear to be focused on affordability.

It is clear that in Indiana, reimbursement could be cut substantially with minimal impact on non-profit hospitals’ long-term financial health. Employers, employees and communities would benefit and, over time, these hospitals would have incentives to confront their high costs.

## Footnotes

1 White (2017).

2 Moody’s Analytics, n.d.

3 Brennan et al. (2017).

4 Hospitals participating in the Medicare program are required to file cost reports each year. The reports are bench audited by CMS. This is publicly available information.

5 As we have several clients in New York, we cannot disclose the location without risking violating non-disclosure agreements (despite the fact the data is in the public domain). We do not have a client in Chicago. Also, by way of disclosure, I was an external board member of American Health Network, an independent medical group headquartered in Indianapolis from 2003 through 2017 when it was sold to Optum.

6 Source: American Association of Health Plans (2016).

7 DRG—Diagnosis Related Group—refers to a standardized system that classifies hospital cases into relatively homogenous groups for the purpose of billing and insurance reimbursement. Most insurers use the same DRG methodology used by Medicare.

8 Indiana University Health System Audited Financial Statements, 2016. IU Health cost reports show a weighted average loss of 13.9%, while their audited financial statements show a gain of 4.9%. Additionally, IU Health took a large charge for exiting certain markets in 2016; this charge appeared on their income statement. When the charge is eliminated, IU Health’s profit margin is 11.9%, consistent with prior years (see Fig. 5). IU Health’s cost reports do not appear to line up with their audited financial statements.

9 Ibid. Rand; Medicare Cost Reports.

10 Stensland et al. (2010).

11 CMS (Centers for Medicare and Medicaid Services 2017).

12 Centers for Medicare and Medicaid Services (2016).

13 United States Census. Quick Facts for Marion County, Indiana and Cook County, Illinois. 2017.

14 Risk Adjustment Factors are derived from a physician evaluation of the patient in a common methodology.

15 This data was supplied by American Health Network, which is an independent physician group that participates in most of the Medicare Advantage programs in the market as well as accountable care. Therefore, we do not expect any statistical bias in their data.

16 For a discussion of this complex subject, see Allen (2017).

17 Implants are expensive, and choices are often driven by physician preference, rather than quality or safety (all implants must be FDA approved).

18 Salaries raise an interesting question. It appears that overpayment by commercial insurers allows health systems to pay more as noted here. Does this in fact inflate the wage index Medicare uses for central Indiana increasing Medicare rates too?

19 Source: Indiana Department of Public Health (2017).

20 Russell and Erody (2018).

21 Kane (2017).

22 Cost and Revenue Report (Medical Group Management Association 2016).
